# Study on the Effect of Relaying on Norovirus Reduction from *Crassostrea gigas* Oysters

**DOI:** 10.3390/microorganisms10122389

**Published:** 2022-12-01

**Authors:** Roberta Battistini, Chiara Masotti, Cristiana Maurella, Erica Costa, Mino Orlandi, Mirvana Feletti, Carlo Ercolini, Laura Serracca

**Affiliations:** 1 Istituto Zooprofilattico Sperimentale del Piemonte, Liguria e Valle d’Aosta, 10154 Turin, Italy; 2 Liguria Local Health Unit-ASL 5, Complex Unit of Hygiene of Foods and Animal Origin, 19122 La Spezia, Italy; 3 Dipartimento Agricoltura, Turismo, Formazione e Lavoro Regione Liguria—Settore Politiche Agricole e Della Pesca, 16121 Genoa, Italy

**Keywords:** *Crassostrea gigas*, relay, norovirus, food safety, real-time qPCR

## Abstract

Norovirus (NoV) is the most important cause of seafood-borne gastroenteritis worldwide, mainly associated with the consumption of raw oysters. NoV is often present in oysters that comply with existing control standards for shellfish. Therefore, the improvement of post-harvest treatments and practices can represent one of the main strategies to reduce the incidence of viral diseases related to shellfish. This study aimed to investigate long-term relays for the reduction of NoV levels in live oysters, during the high-risk cold months, by transferring the oysters from a more contaminated site to two sites with lower NoV levels. The efficacy of relaying was evaluated by analyzing oyster samples collected at days 0 (T0) and 30 (T30) for NoV levels using a real-time quantitative PCR (RT-qPCR). The NoV level at the relay sites was consistently lower than at the harvest site. The relay process for 30 days in seawater with a lower NoV level resulted in a decrease in the NoV load compared to day 0 with significant reductions depending on the site and genogroup of NoV considered. These results suggest that long-term relaying of oysters to reduce NoV levels is promising and could help growers to improve oyster safety; however, further investigations are needed.

## 1. Introduction

Oysters are filter-feeding organisms capable of concentrating pathogenic microorganisms from the environment in which they live. The widespread habit of consuming these raw shellfish exposes the consumer to the risk of gastroenteritis. Among the main pathogens responsible for foodborne gastroenteritis are Noroviruses (NoVs) which caused 130 outbreaks in Europe in 2020 [1]. The latest European Food Safety Authority (EFSA) study in 2021, which measured NoV contamination in raw oysters, reported that one-third of mussels in Europe are contaminated. NoV was found in 34.5% of mussels collected at production sites and in 10.8% of those for sale. The survey showed higher NoV contamination in the period from November to April, as well as lower contamination for class A areas compared to class B and class C areas. In the class A areas, the laws do not provide for particular treatments before marketing, which is necessary for oysters from the class B areas [2]. NoV infection is prevalent from November to April and is sometimes called “winter vomiting disease” or “stomach flu”. The virus is highly infectious, and ten viral particles are enough to give rise to an infection [3]. NoV is transmitted mainly through the fecal–oral route, by consumption of contaminated food or water, or directly from person to person, and also by contact with contaminated surfaces. NoV can be excreted in high levels (up to 10^9^ viruses/g feces) in the feces of infected individuals [4]. Therefore, during the winter period, high concentrations of NoV can be found in wastewater [5]. Wastewater discharge into aquatic environments is practiced worldwide, representing an important issue in coastal seawaters during the winter and leading to the contamination of bivalve mollusk production areas. In particular, oysters contaminated with NoV pose an important risk to human health since they are usually consumed raw [6].

The conventional approach to the purification of oysters envisaged by current legislation is depuration, a process used to remove microorganisms and other contaminants from bivalve mollusks by placing them in tanks of clean seawater, often recycled and disinfected using ultraviolet light, ozone, or other means [3]. Depuration is used worldwide, and the depuration periods are varied from a few hours to several days, depending on the country [7]. The effectiveness of the depuration process is evaluated by the ability to reduce the bacterial count, often using fecal coliform bacteria and, in particular, the “target” microorganism *E. coli*. Depuration of shellfish is not effective for reducing enteric viruses, and episodes of viral gastroenteritis associated with the consumption of bivalve mollusks evaluated with the *E. coli* parameter occur annually around the world [8]. It is therefore important to identify different post-harvest intervention strategies to reduce these pathogens in oysters in order to increase their safety for consumers [9].

Relaying is an alternative treatment to depuration; this involves a longer-term purification process (often requiring ten days or longer) [10]. In relaying, bivalve mollusks are collected from a contaminated area and transferred to pollution-free marine environments, where they are maintained for a shorter or longer period to allow them to purge contaminants derived from wastewater under natural environmental conditions.

Limited data on the effectiveness of prolonged relaying for NoV reduction are currently available [11,12,13,14], and further studies about this topic are needed, preferably using the standardized European Committee for Standardization (CEN) method [9]. So far, two studies have suggested that a relaying period in seawater of around four weeks may be sufficient to reduce NoV levels below the limit of quantification (LOQ) in oysters [11,13]. Unfortunately, very few marine areas are completely NoV-free, and also the waters classified as class A are contaminated by NoV. Therefore, our study evaluated the reduction of NoV concentration in the winter months using the quantitative real-time RT-PCR method to further investigate the long-time relaying period by relaying oysters for 30 days in seawater sites with less NoV contamination.

## 2. Materials and Methods

### 2.1. Oyster Sampling Sites

**Figure 1:**
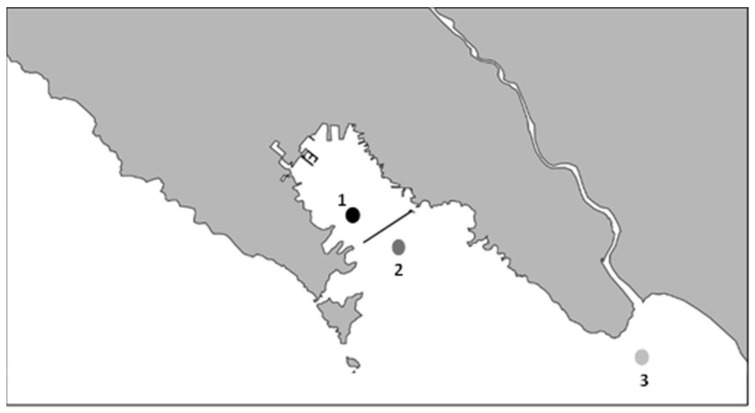
Schematic map of sampling sites: (1) Main production area, (2) relaying site 2, (3) relaying site 3. The approximate distance from (1) to (2) is 0.94 Km, and (1) to (3) is 11.5 Km.

Oysters (*Crassostrea gigas*) used in this study were harvested from a class B shellfish farming area in northwestern Italy (site 1) and moved to two different sites of category A (site 2 and site 3) (Figure 1). Site 1 is located inshore in a very anthropized area characterized by a commercial and tourist port and shipbuilding activity. Sites 2 and 3 are offshore at approximately 1.6 km and 1.8 km from the coast, respectively (Figure 1). Sixty oysters were harvested from a single sampling point in the main production area (site 1) every month, for a total of four samplings, during the coldest months of the year (November–February) when NoV concentration is supposed to be the highest. Twenty oysters were immediately analyzed (ten individuals tested twice) for NoV genogroup I (GI) and genogroup II (GII) (T0) from each sampling. The other oysters were moved to site 2 and site 3 with less NoV contamination and left for relaying in these seawaters for one month. After 30 days, the 20 oysters from site 2 and the 20 oysters from site 3 were collected, transferred to the laboratory in refrigerated condition, and immediately analyzed for NoV GI and GII (10 individuals tested twice for each site) (T30).

Additionally, samples of 20 oysters were harvested from sites 2 and 3 on the same days as the oyster sampling at site 1 (T0). Further samples of 20 oysters were sampled from sites 2 and 3 on the same days as the oyster sampling after 30 days of relaying (T30). These additional samples were analyzed for NoV GI and NoV GII to monitor the concentration of NoV in native oysters. Seawater parameters, such as temperature and salinity, were recorded at each sampling in site 2 and 3.

### 2.2. Virus Recovery from Oysters

Oyster samples (each composed of ten oysters) were tested according to the ISO 15216-1:2017 method [15]. The hepatopancreas (on average 5 g per oyster) was removed by dissection from each oyster, pooled, and homogenized with TissueLyser (Qiagen, Hilden, Germany). A total of 10 µL of Mengovirus (process control virus) and 2 mL of proteinase K (0.1 mg/mL) were added to 2 grams of homogenates. The homogenates were incubated for 60 min at 37 °C with shaking at 320 rpm and, after that, maintained at 60 °C for 15 min in the water bath and centrifuged for 5 min at 3000× *g*. Finally, supernatants were recovered for RNA extraction, and their volumes were recorded.

### 2.3. RNA Extraction

Viral RNA was extracted from 500 µL of the supernatants using the EGENE-UP^®^ platform and the NucliSens magnetic extraction reagents (bioMérieux, Marcy l’Etoile, France) according to the manufacturer’s instructions. RNA was eluted into 100 μL of elution buffer and was immediately used for the NoV quantification by real-time RT-PCR or stored at −80 °C until real-time RT-PCR analysis.

### 2.4. Quantification of Norovirus by Real-Time RT-PCR

The detection and quantification of NoV GI and NoV GII genomes were performed by real-time quantitative reverse transcription PCR (RT-qPCR) according to ISO 15216-1:2017, and the reactions were carried out using the Biorad CFX96TM Real-Time PCR thermocycler (Biorad, Hercules, CA, USA). The number of RNA copies per μL of each sample was calculated by matching the sample Cq value to the standard curves (one for each target) created with the tenfold serial dilution of a dsDNA standard for NoV GI and NoV GII. Therefore, the final concentrations were expressed as genomic copies per gram (g.c./g) and were calculated based on the volume of the analyzed extract. According to ISO 15216-1:2017, the Cq value of Mengovirus was obtained in spiked samples and was compared with extracted samples by viral stock to evaluate extraction efficiency. Furthermore, to evaluate the inhibition of RT-qPCR, the Cq value was obtained in samples spiked with 1 µL of external control RNA for both GI and GII and was compared with that obtained in samples without the addition of external control. Results with extraction efficiency > 1% and RT-qPCR inhibition ≤ 75% were considered valid. The LOQ was established by the European Union Reference Laboratory (EURL). The LOQ for NoV GI was calculated as 140 g.c./g and 130 g.c./g for NoV GII.

### 2.5. Statistical Analysis

Data were verified for Normality using the Shapiro–Wilk test. The Wilcoxon rank-sum test was used to compare the samples between the two oyster farming areas at time T0. The Wilcoxon matched-pairs signed-rank test was used to compare the equality of matched pairs of observations, i.e., time T0 versus T30.

## 3. Results

**Figure 2:**
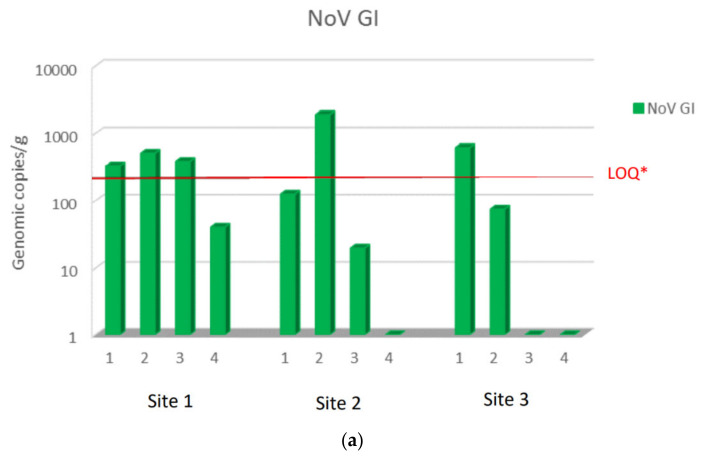

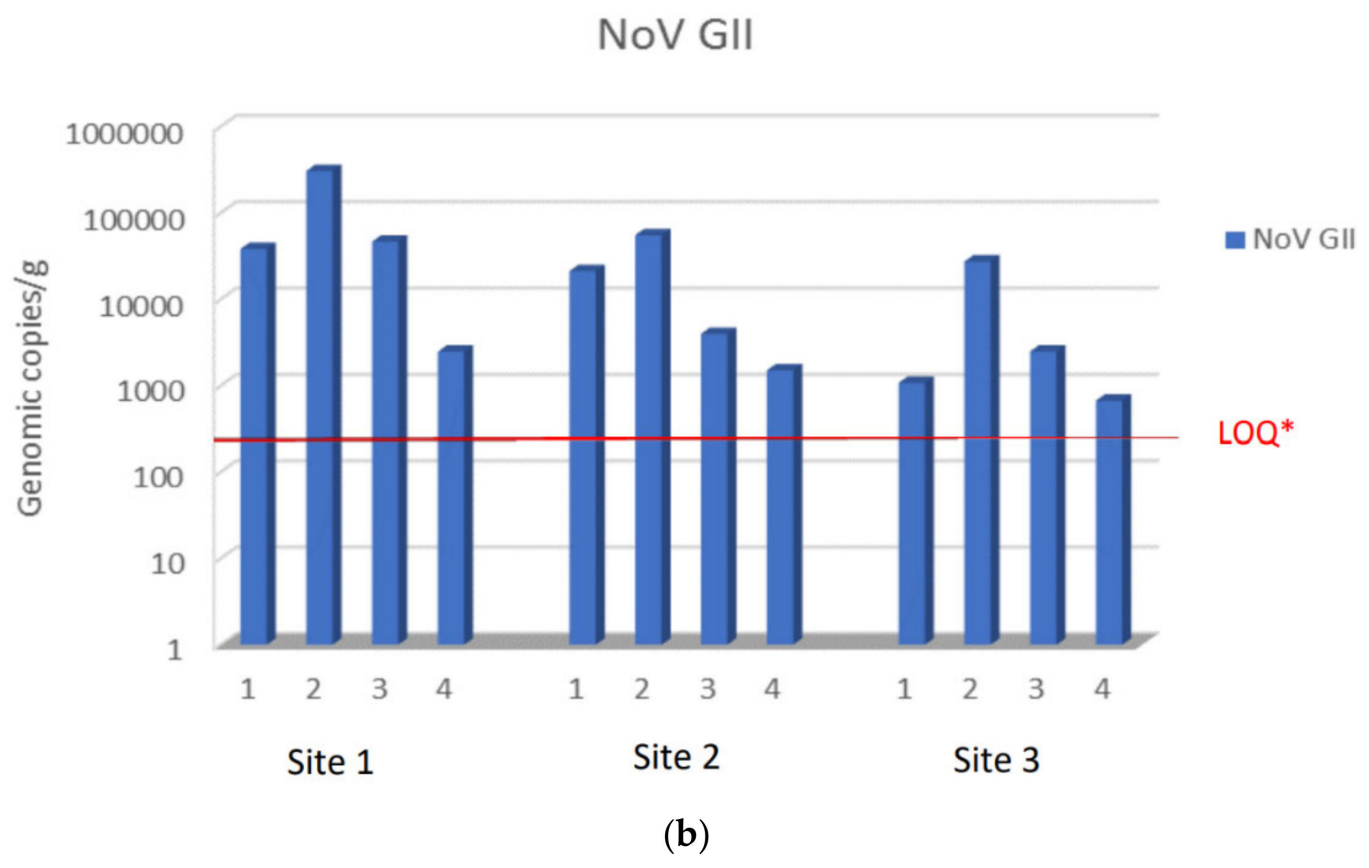
NoV RNA levels detected in oysters at Time 0 of the relay (T0) at site 1, site 2, and site 3: (**a**) NoV GI; (**b**) NoVGII. Values are expressed in genome copies/gram. * LOQ: 140 g.c./g for NoV GI and 130 g.c./g for NoV GII.

During the study period from November to February, NoV GI and NoV GII were always detected at harvest site 1 in a concentration higher than sites 2 and 3, except for NoV GI in December month (second sampling), when it was present at higher concentration at site 2. NoV GII was found at all sites at higher concentrations than NoV GI. The maximum NoV GII load was 3.1 × 10^5^ viral g.c./g at site 1, 5.5 × 10^4^ viral g.c./g at site 2, and 2.7 × 10^4^ viral g.c./g at site 3. The maximum load of NoV GI was 5.1 × 10^2^ viral g.c./g at site 1, 1.9 × 10^3^ viral g.c./g at site 2, and 6.2 × 10^3^ viral g.c./g at site 3. Moreover, NoV GII was always detected in all sampling, while NoV GI was absent or below the LOQ in some samples at sites 2 and 3. Among the sites used for relaying, site 3 was the one with a lower concentration of both NoV GI and NoV GII compared to site 2 (Figure 2a,b).

**Figure 3:**
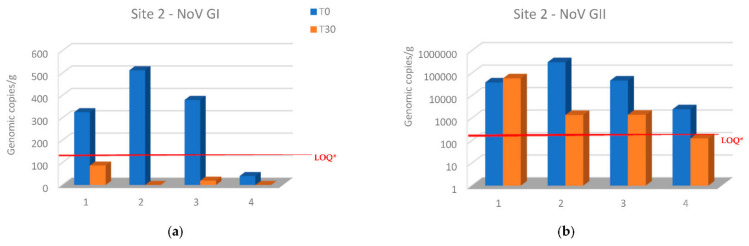
Comparison of NoV RNA levels in oysters at Time 0 of the relay (T0) and after 30 days of the relay (T30) at site 2: (**a**) NoV GI; (**b**) NoV GII. Values are expressed in genome copies/gram. * LOQ: 140 g.c./g for NoV GI and 130 g.c./g for NoV GII.

NoV GI levels decreased in all samples below the LOQ of the assay (140 genomic copies per g) at site 2 after 30 days from the transfer of oysters from site 1. Moreover, NoV GI was not detected in two samples after relaying period (Figure 3a). In the same site, NoV GII levels decreased in all oyster samples except in the first sampling, while NoV GII levels decreased below the LOQ (130 genomic copies per g) in the last sampling (Figure 3b).

**Figure 4:**
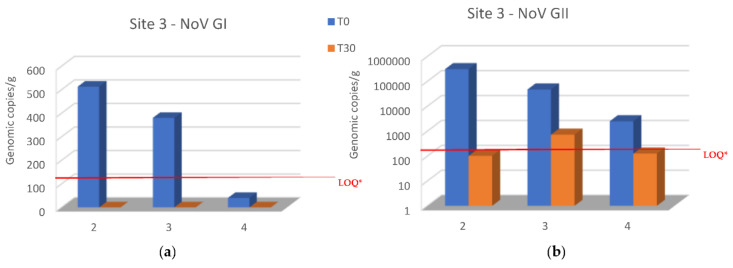
Comparison of NoV RNA levels in oysters at Time 0 of the relay (T0) and after 30 days of the relay (T30) at site 3: (**a**) NoV GI; (**b**) NoV GII. Values are expressed in genome copies/gram. * LOQ: 140 g.c./g for NoV GI and 130 g.c./g for NoV GII.

Both NoV GI and NoV GII levels decreased in all oyster samples of site 1 when transferred to site 3 after 30 days of relaying (Figure 4a,b), and NoV GI was no longer detected after this period (Figure 4a). NoV GII was below the LOQ in samples collected in the second and fourth sampling (December and February) (Figure 4b). Unfortunately, it was not possible to evaluate the samples placed at site 3 in November because the samples were not found after 30 days, probably due to theft by unknown persons.

**Figure 5:**
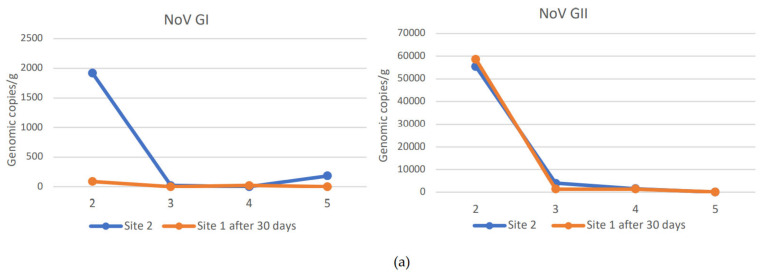

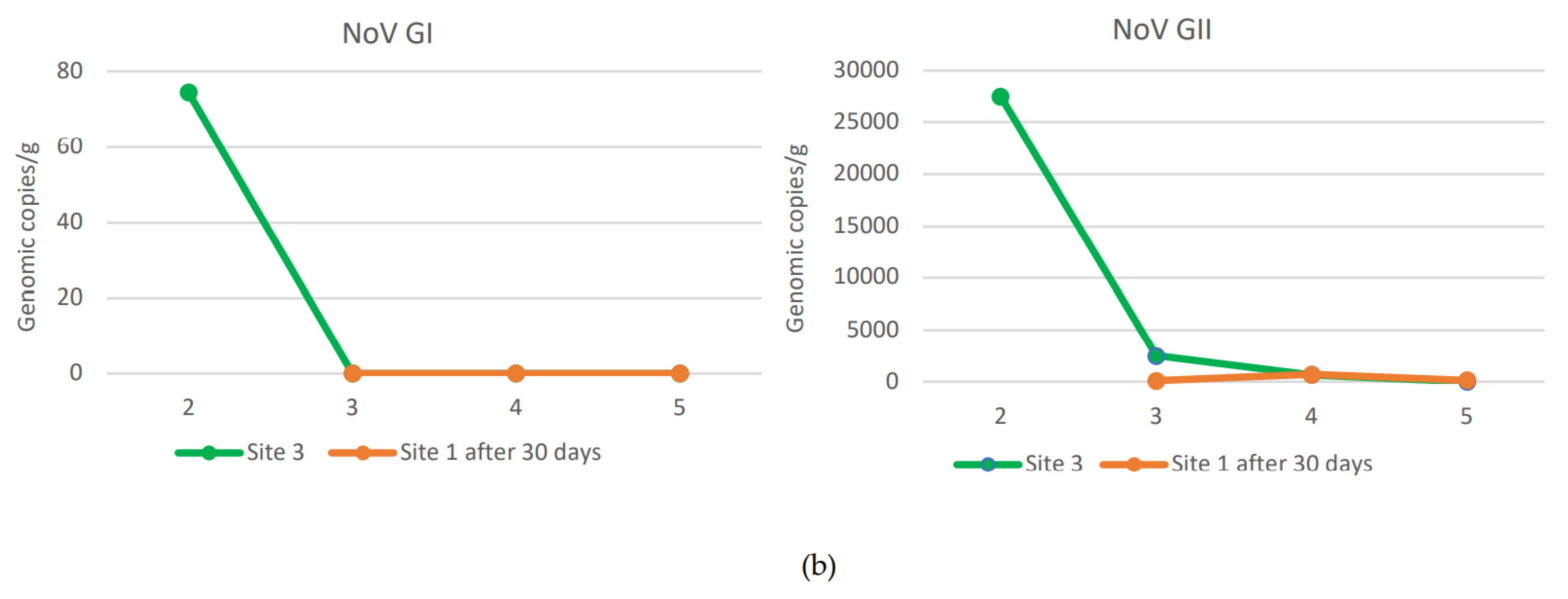
NoV GI and NoV GII values in oysters at site 1 after 30 days of relaying at site 2 (**a**) or site 3 (**b**). NoV GI and NoV GII values in oysters naturally presented at site 2 (**a**) or site 3 (**b**) at T30.

Relays for 30 days in seawater with a lower NoV level resulted in a decrease in the NoV load in oysters compared to T0 at both sites 2 and 3. In particular, logarithmic reductions between 2.34 and −0.2 and between 2.7 and 0.56 were obtained, respectively, for NoV GII and NoV GI at site 2 (Figure 3). Although, log reductions ranged from 3.48 to 1.3 for NoV GII and from 2.7 to 1.6 for NoV GI at site 3 (Figure 4). After 30 days of relaying, the load of NoV in oysters usually tends to conform to the area where they are introduced (Figure 5). Based on statistical analysis, no difference between the two groups was found at time T0, but a statistically significant difference was found by comparing the withdrawal data both for NoV GI (*p* = 0.04) and for Nov GII (*p* = 0.009). A statistically significant difference was found by comparing the paired data between time T0 and time T30 both for NoV GI (*p* = 0.001) and for Nov GII (*p* = 0.01). The comparison between the two consecutive summations also gave a significant result (*p* = 0.01). The statistical analysis of stratified sampling at relaying area gave the following results: at site 2, only the reduction of NoV GI was significant (*p* = 0.031), while at site 3, both NoV GII and the sum of NoV GI and NoV GII reduction were significant (*p* = 0.031).

Physiochemical parameters of seawater during oysters’ relay periods are summarized in Table 1. Temperature and salinity showed no significant variations between the two sites in the same months.

## 4. Discussion

In this study, we report the results of prolonged relaying to decrease NoV concentration in oysters as a possible management strategy to reduce consumer exposure to NoV in the winter season. To study NoV reduction, we used environmentally contaminated oysters from approved production areas classified as Class B areas and transferred them to two different sites with less contamination classified as Class A during the high-risk winter period. NoV monitoring at the main production sites (site 1) and the relaying sites (sites 2 and 3) confirmed that oysters at the main production site contained, on average, higher NoV concentrations.

In our study, the presence of high levels of NoV at site 1, compared to sites 2 and 3, is probably related to the characteristics of the area where the oysters are raised. The oyster farm located at site 1 is inside a dam, which protects the commercial port from the open sea by reducing sea currents and favoring the stagnation of contaminants in this area. Whereas in oyster farms located at sites 2 and 3, the depth of the seabed and the distance from the coast favor the dilution and quantity of contaminants present in marine waters and reduce the risk of contamination of oysters.

The data presented here indicate that oysters from the main collection site significantly reduced their NoV concentration by relaying for 30 days in seawater sites with a lower NoV concentration. In only one case, the NoV GII concentration increased after 30 days at site 2 (Figure 3b). The increased concentration can be explained by analyzing the NoV GII level found in the second sampling in this area. In fact, when sample 1 was placed at site 2 (T0) in November, the NoV concentration was lower than that present at the re-immission site. During the following 30 days, the NoV load at site 2 increased, as seen in Figure 2b. Therefore, in this case, the sample moved from site 1 to site 2 could not reduce the NoV load present in it during the relaying period. Another unexpected result is related to the first sample from site 1 introduced into site 2, containing after 30 days a lower load of NoV GI than the oysters always present at site 2 (Figure 5a). These data are related to the second sampling carried out in December, when evidently there was a peak of both NoV GI and NoV GII in this area. This case highlights a different behavior of the two NoV genogroups in oysters, which bind to different ligands within the tissues of the oysters and, therefore, are concentrated and/or eliminated differently, as already highlighted in other studies [16,17]. Except for this case, the NoV values after the relaying period tend to conform to those values recorded in oysters already present at sites 2 and 3 (Figure 5). Indeed, the best NoV reduction value was obtained in the oyster samples after 30 days at site 3, which maintained a lower NoV load during the winter period. In detail, in 67% of the oyster samples, NoV was <LOQ at site 3, while at site 2, only 25% of the samples were below the LOQ. NoV GI was reduced at both sites in all samples below the LOQ, while the NoV GII was reduced more at site 3, with 50% of the samples < LOQ after 30 days of relays in adherence to the situation present in the relaying areas. No threshold infectivity limit is currently established for NoV as detected by polymerase chain reaction (PCR). However, it is shown that a low probability of outbreaks is associated with oysters containing NoV in concentration levels below 152 g.c./g., which in our study are present in samples below the LOQ. However, it is not possible to conclusively rule out the possibility of oysters containing levels of <152 c.g./g. causing illness, and it seems likely that these levels present a comparatively lower risk. In addition, there is some indication that at higher levels (>500 c.g./g.), the risk becomes greater [18].

So far, few studies have reported a significant reduction in NoV load from oysters relayed in seawater over an extended period. In one case, oysters from the harvesting area responsible for NoV outbreaks were moved to a seawater site free from sewage contamination and maintained there for 17 days [13]. In another study, the oysters reduced NoV concentration by <500 genomic copies/g in all samples relayed during the winter season in an alternative site with less NoV contamination. In contrast, 31% of oyster samples kept at the native harvest site contained NoV > 500 genomic copies/g [14].

The ideal procedure for obtaining oysters safely would be harvesting them in areas that are not subjected to any type of contamination. However, very few such areas really exist, and also the waters classified as class A are contaminated by NoV. Moreover, access to clean relaying waters may be a challenge in the future, with an increasing global population. To overcome these problems, long-term relaying could be a mitigation strategy for the enteric virus reduction; if possible, move the oysters from areas with high levels of NoV to areas with a lower viral concentration in order to reduce the risk to consumers as much as possible. Indeed, in some studies, it is observed that the likelihood of becoming infected with NoV increases with NoV dose [4,19,20,21]. Furthermore, a correlation was found between the amount of self-reported disease and the number of copies of the NoV genome in oysters [18,22]; higher concentrations of NoV RNA correspond to a higher rate of reported diseases, suggesting a link between virus RNA levels and health risks. Currently, the most widespread method to reduce contamination is depuration, with good results in the elimination of fecal bacteria but scarcely or not all effective in eliminating pathogenic viruses. The other post-harvest treatments, such as frozen storage, thermal inactivation, and high-pressure processing, also require either a significant amount of initial investment or operation costs and often change the organoleptic characteristics of shellfish, making them unacceptable to consumers [23]. The application of long-term relaying instead could provide a practical, less expensive, and natural alternative to other methods by enabling the reduction of NoV concentrations to levels that reduce, if not eliminate, the risk to consumers. Long-term relaying is especially important for producers that can move the oysters to less contaminated sites close to other more contaminated production areas. Considering that the oyster takes 18 to 24 months to become an adult or reach market size (about 3 inches), it would be a matter of moving oysters to the relaying sites one month before being sold, in the winter season only, so to minimize the extra costs related to the relocation process.

In our study, the obtained dataset provides statistically significant differences between the different sites studied and between the different genogroups and gives an initial indication of the trends and effects of this treatment. However, further studies during winter seasons may be useful to integrate the data presented here. The limitation of our study was the lack of Norovirus-free marine areas to move the oysters to for relaying since this strategy would be the best for studying the oyster relaying. Unfortunately, in our case, no NoV-free marine areas are close to the main production site where the oysters could be transferred at affordable costs before sale. As discussed above, it is increasingly difficult to find NoV-free marine waters, and, as in our case, waters classified in class A are also usually contaminated by NoV, albeit in a lower concentration than the sites classified in class B. Therefore, in our study, we chose to move the oysters from a more NoV-contaminated site to less NoV-contaminated sites in order to study the long-term relay for the reduction of NoV concentrations in oysters.

## 5. Conclusions

In conclusion, the results of this study suggest that relaying naturally contaminated oysters to sites close to the main production area with lower NoV concentration before their final harvest could provide a practical and low-cost mitigation strategy to reduce NoV in oysters. Relaying could lead to a decrease in the risk of NoV disease from oyster consumption during the colder season when NoV is present in high concentrations. This study may help growers to improve both the marketability and safety of oysters, as well as provide additional information relevant to risk management decisions for regulatory agencies in light of the potential introduction of a statutory limit.

## Figures and Tables

**Table 1:** Physiochemical parameters of seawater measured during oysters’ relaying period at sites 2 and 3.

	Site 2		Site 3	
	T (°C) Media ± dev. st.	Salinity (PSU)	T (°C) Media ± dev. st.	Salinity (PSU)
November	17.0 ±1.4	37.5 ± 0.5	17.2 ± 1.2	36.7 ± 0.7
December	13.6 ±1.1	37.3 ± 0.6	12.6 ± 1.0	36.2 ± 0.5
January	13.8 ±1.0	37.8 ± 0.4	12.4 ± 0.6	36.7 ± 0.4
February	12.8 ± 0.5	37.8 ± 0.6	12.9 ± 1.1	37.5 ± 0.5
March	13.4 ± 0.85	38.0 ± 0.5	13.5± 0.9	37.6 ± 0.6

## Data Availability

Not applicable.
